# Maternal illnesses during pregnancy and the risk of childhood cancer: A medical‐record based analysis (UKCCS)

**DOI:** 10.1002/ijc.35166

**Published:** 2024-11-13

**Authors:** Audrey Bonaventure, Jill Simpson, Eleanor Kane, Eve Roman

**Affiliations:** ^1^ Epidemiology of Childhood and Adolescent Cancer (EPICEA) Team, Center for Research in Epidemiology and StatisticS (CRESS) Université Paris Cité, Université Sorbonne Paris Nord, INSERM, INRAE Villejuif France; ^2^ Epidemiology and Cancer Statistics Group, Department of Health Sciences University of York York UK

**Keywords:** anaemia, childhood cancer, infection, medical record, pregnancy

## Abstract

Often relying on mother's recollections of past events, the possible relationship between maternal illness in pregnancy and risk of malignancy in their offspring has long been a focus of research. Free from recall bias, this study of childhood cancer (0–14 years) examined these associations using data abstracted from mothers' primary‐care (1623 cases, 2521 controls) and obstetric (2721 cases, 5169 controls) records. Maternal infections and other illnesses in pregnancy were examined for any possible associations with childhood leukaemia, lymphoma, CNS or embryonal tumours using pooled information from the two medical record sources (2885 cases and 5499 controls), accounting for potential confounders. Maternal anaemia was associated with childhood acute myeloid leukaemia (AML) (odds ratio, OR = 2.07, 95%CI [1.40–3.08]). Anaemia during pregnancy was also recorded more frequently in the notes of mothers of children with medulloblastoma, retinoblastoma and embryonal rhabdomyosarcoma: ORs 2.36 [1.36–4.11], 1.83 [1.01–3.33] and 2.91 [1.64–5.16] respectively. Other associations included urinary tract infections (UTIs) and non‐Hodgkin lymphoma (NHL); preeclampsia and NHL; and polyhydramnios with both AML and NHL. No evidence was found to suggest that influenza during pregnancy impacted on childhood leukaemia risk. In conclusion, our findings are supportive of an association between maternal anaemia in pregnancy and childhood AML, and maternal anaemia and embryonal tumours; underscoring the need for further research exploring the potential causes and roles of iron and vitamin deficiencies. Due to small numbers and lack of corroborative evidence, the associations observed for UTIs, preeclampsia, and polyhydramnios must be treated cautiously.

## What's new?

Relationships between maternal illness during pregnancy and cancer risk in offspring have been investigated for decades, with inconsistent results. Here, the authors reviewed medical records to examine relationships between maternal illness in pregnancy and several types of childhood cancer. Analyses reveal associations between maternal anaemia in pregnancy and childhood acute myeloid leukaemia and certain embryonal tumours. No evidence was found, however, linking maternal infections to increased risk of childhood cancer, with the exception of non‐Hodgkin lymphoma. The findings warrant additional exploration of the impact of maternal anaemia, iron and vitamin deficiency in pregnancy on the pathogenesis of childhood leukaemia and embryonal tumours.

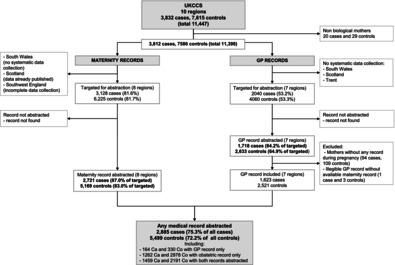

## 1 INTRODUCTION

Since Norman Gregg first reported an association between rubella infection in pregnancy and congenital cataracts in 1941, research into the relationship between maternal illnesses in pregnancy and child health has proliferated. In particular, a number of studies have reported positive associations between maternal infections in pregnancy and childhood leukaemia and other cancer types.^1^, ^2^, ^3^, ^4^, ^5^, ^6^, ^7^, ^8^, ^9^, ^10^ Albeit inconsistently, positive associations have also been reported with maternal anaemia,^11^, ^12^, ^13^, ^14^ hypertensive disorders including preeclampsia,^1^, ^15^, ^16^, ^17^, ^18^, ^19^, ^20^, ^21^, ^22^, ^23^ and maternal diabetes (recently reviewed^24^, ^25^). Polyhydramnios and morning sickness/hyperemesis have also been investigated. However, findings have not been consistent across studies, and questions on maternal infections and other illnesses in pregnancy remain. Perhaps the primary reason for inconsistencies is that most studies have been based on information about health events reported by mothers several years after they occurred; providing the potential for recall bias. Indeed, the reliability of questionnaire‐reports of illnesses and drug intake during pregnancy is of major concern for interview‐based epidemiological studies.^26^, ^27^ The present report uses data from medical records abstracted in England and North Wales during the course of a national childhood cancer case‐control study.

## 2 MATERIALS AND METHODS

The United Kingdom Childhood Cancer Study (UKCCS) is a national population‐based case‐control study established in the 1990s to investigate the potential causes of childhood cancer and details of its conduct and ethical approvals have been fully described elsewhere.^28^, ^29^, ^30^, ^31^ Briefly, the study population comprised all children (0–14 years) registered for primary care with the National Health Service (NHS) in England, Scotland and Wales. All children newly diagnosed with cancer (cases) were ascertained via proactive notification systems established in all treatment centres; and each child whose parents agreed to be interviewed (87%) was individually matched on sex and age (month and year of birth) to up to 10 randomly selected controls. The general practitioners (GPs) of the first two controls identified were approached and, with their permission, the parents of the children were contacted and asked to participate in the study. Overall, 72% of these first‐choice control families participated; but if the GP refused permission to contact the parents, or the parents themselves declined, the next control on the list was selected, and so on until two control families per case had agreed.

**FIGURE 1:**
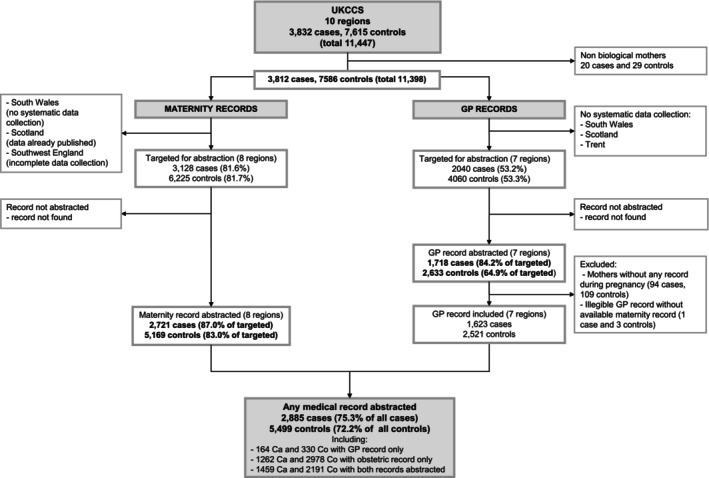
Flowchart of the study participants.

The flowchart in Figure 1 shows the derivation of the study numbers from mothers who were interviewed through to those who had medical record data that contributed to this analysis. At interview, mothers were asked for consent to access their medical notes. Of the 3832 case and 7615 control families living in one of the 10 UKCCS administrative areas and enrolled in the study, consent to access medical records was not obtained from the biological mothers of 20 cases and 29 controls (children were adopted); leaving 3812 and 7586 cases and controls potentially eligible for maternal medical record abstraction^29^, ^30^ The present analyses were restricted to children residing in England and North Wales for whom maternal medical record information was available from either (1) obstetric records, which were abstracted for 2721 case mothers and 5169 control mothers, or (2) primary‐care records, which were abstracted for 1718 case mothers and 2633 control mothers. After excluding incomplete records, a total of 1623 cases and 2521 controls with information from maternal GP notes were available for analyses. In total, medical records (GP and obstetric combined) were available for 2885 case mothers and 5499 controls mothers (75.3% all cases and 72.2% all controls).

Information was abstracted onto structured forms (accessible at www.ukccs.org/) using standardized operating procedures, and data were coded to the International Statistical Classification of Diseases and Related Health Problems, Tenth Revision (ICD‐10). All medical events that occurred between the date of the last menstruation up to the day before delivery were examined, as well as pre‐existing diabetes, which could have been recorded at any time before the index child's birth. Haemoglobin levels were systematically abstracted from obstetric record. ICD‐codes were grouped into infections and specified non‐infectious conditions (see Supplementary Table 1). Mothers were considered to be anaemic if it was recorded in the medical records on at least one occasion, or if at least one haemoglobin level recorded during pregnancy was <10 g/dL. Area‐based deprivation scores were derived from the area of residence at birth and data from the national 1991 census, using standard methods.^28^

Odds ratios (ORs) and 95% confidence intervals (95%CI) were computed using polychotomous logistic models, adjusting for the year of birth, the child's age at diagnosis (pseudodiagnosis for controls), and an indicator for whether maternal GP record data were available or not. Inclusion of sex made no difference to the findings, so is omitted from the models presented here. Two outcome variables were created using either broad cancer types (leukaemia, lymphoma, CNS tumours, embryonal tumours or other), or more specific subtypes (ALL, AML, NHL, HL, etc.). For each cancer type, the lowest recorded haemoglobin level during pregnancy was also analysed as a continuous variable, and departure from linearity was assessed using likelihood ratio tests comparing two models based on categorical and continuous variables.

Sensitivity analyses were performed excluding children with Down syndrome (3 controls, 22 ALL cases, and 12 AML cases); with additional adjustment for maternal age at birth, pregnancy order, UKCCS administrative region and deprivation quintile; and using a broader haemoglobin 11 g/dL threshold for the definition of anaemia. Analyses were performed using the SAS 9.4 software (SAS Institute Inc., Cary, NC, USA). ORs are not shown when the number of exposed children was below five in a stratum.

## 3 RESULTS

### 3.1 Description of the study participants

Table 1 shows the characteristics of the children included in the present analysis. The distributions by sex, age, maternal age at child's birth, pregnancy order and deprivation index quintiles were generally similar in cases and controls. Slight differences were observed between those with GP information and the whole study with respect to child's age and deprivation quintile, in similar proportions for cases and controls (Supplementary Table 2). The distribution of cancer groups was broadly similar by type of record abstracted; the slightly higher frequency of leukaemia cases in those with maternal information from primary care reflects the emphasis placed on obtaining medical records for leukaemia cases (children and parents) and their corresponding controls in certain UKCCS regions.^30^

**TABLE 1:** Description of the UKCCS participants with maternal medical records abstracted (primary care and/or obstetric records).

	Controls (*N* = 5499) *N* (%)	Cases (*N* = 2885) *N* (%)
Sex		
Female	2431 (44.2)	1274 (44.2)
Male	3068 (55.8)	1611 (55.8)
*Age of child at diagnosis/pseudodiagnosis* (years)		
1st year of life	490 (8.9)	256 (8.9)
1–4	2402 (43.7)	1242 (43.1)
5–9	1517 (27.6)	794 (27.5)
≥10	1090 (19.8)	593 (20.6)
Mean (SD)	5.3 (4.06)	5.4 (4.1)
*Maternal age at birth* (years)		
<25	1617 (29.4)	934 (32.4)
25–29	2015 (36.6)	1057 (36.6)
>29	1867 (34.0)	894 (31.0)
Mean (SD)	27.5 (5.15)	27.0 (5.15)
*Pregnancy order*		
1	1885 (34.3)	1004 (34.8)
2	1815 (33.0)	934 (32.4)
3	993 (18.1)	515 (17.9)
4+	806 (14.7)	432 (15.0)
*Deprivation quintile*		
(Most affluent) 1	1027 (18.7)	506 (17.5)
2	1152 (20.9)	611 (21.2)
3	1230 (22.4)	600 (20.8)
4	1111 (20.2)	586 (20.3)
(Most deprived) 5	952 (17.3)	566 (19.6)
Missing	27 (0.5)	16 (0.6)
*Cancer type*		
Leukaemia		1347 (46.7)
Lymphoma		254 (8.8)
CNS tumour^a^		510 (17.7)
Embryonal tumour^a^		446 (15.5)
Other		328 (11.4)

^a^ Medulloblastoma included in the group of CNS tumours.

### 3.2 Associations with haematological malignancies

Having at least one record of an infection during pregnancy was positively associated with lymphoma but no support for an association with leukaemia was observed (Table 2). The association with lymphoma was partly driven by the relationship between urinary tract infections and non‐Hodgkin lymphoma (NHL) (OR = 2.36 [1.46–3.81]) (Table 3). Influenza and chickenpox were rarely diagnosed in pregnancy (1.2% and 0.4% of control mothers, respectively), and no evidence of associations between influenza and childhood leukaemia overall (adj. OR = 1.04 [0.64–1.69]) or ALL in particular (22 cases exposed, OR = 1.22 [0.74–1.99]) was observed. Likewise, there was no evidence supporting an association between varicella and childhood leukaemia.

**TABLE 2:** Associations between maternal illnesses in pregnancy and childhood cancer, by main cancer types.

	Controls, *N* = 5499	Leukaemia, *N* = 1347		Lymphoma, *N* = 254		CNS tumour, *N* = 510		Embryonal tumour, *N* = 446		Other cancer types, *N* = 328	
	*N* (%)	*N* (%)	OR [95% CI]	*N* (%)	OR [95% CI]	*N* (%)	OR [95% CI]	*N* (%)	OR [95% CI]	*N* (%)	OR [95% CI]
*Infections*											
Any infection	1546 (28.1)	454 (33.7)	0.95 [0.83–1.09]	77 (30.3)	1.37 [1.02–1.85]	143 (28.0)	1.06 [0.85–1.31]	137 (30.7)	0.94 [0.75–1.18]	85 (25.9)	0.98 [0.75–1.28]
Urinary tract	319 (5.8)	86 (6.4)	0.98 [0.76–1.26]	25 (9.8)	1.95 [1.26–3.02]	31 (6.1)	1.06 [0.72–1.56]	26 (5.8)	0.88 [0.58–1.34]	20 (6.1)	1.09 [0.68–1.75]
Genital	268 (4.9)	79 (5.9)	0.93 [0.71–1.21]	9 (3.5)	0.77 [0.39–1.54]	19 (3.7)	0.75 [0.47–1.22]	23 (5.2)	0.87 [0.55–1.36]	17 (5.2)	1.09 [0.66–1.83]
Influenza	68 (1.2)	23 (1.7)	1.04 [0.64–1.69]	5 (2.0)	1.58 [0.62–4.03]	4 (0.8)	—	9 (2.0)	1.72 [0.84–3.53]	3 (0.9)	—
Chickenpox	23 (0.4)	6 (0.4)	0.84 [0.34–2.10]	0 (0)		2 (0.4)	—	4 (0.9)	—	0 (0)	—
*Other illnesses*											
Gestational HT or preeclampsia	533 (9.7)	138 (10.2)	1.08 [0.88–1.32]	33 (13.0)	1.36 [0.93–2.00]	56 (11.0)	1.16 [0.86–1.55]	37 (8.3)	0.87 [0.61–1.24]	31 (9.5)	0.98 [0.67–1.43]
Preeclampsia	139 (2.5)	34 (2.5)	1.03 [0.70–1.52]	13 (5.1)	2.03 [1.12–3.67]	17 (3.3)	1.33 [0.79–2.23]	14 (3.1)	1.26 [0.71–2.24]	9 (2.7)	1.08 [0.54–2.15]
Polyhydramnios	53 (1.0)	20 (1.5)	1.67 [0.98–2.84]	5 (2.0)	1.99 [0.77–5.10]	7 (1.4)	1.37 [0.62–3.04]	9 (2.0)	2.08 [1.00–4.33]	5 (1.5)	1.53 [0.60–3.87]
Any vomiting or hyperemesis	743 (13.5)	203 (15.1)	0.95 [0.80–1.13]	34 (13.4)	1.08 [0.74–1.57]	74 (14.5)	1.09 [0.84–1.42]	76 (17.0)	1.17 [0.90–1.53]	47 (14.3)	1.10 [0.79–1.52]
Diabetes	59 (1.1)	13 (1.0)	0.88 [0.47–1.63]	4 (1.6)	—	5 (1.0)	0.97 [0.38–2.43]	3 (0.7)	—	2 (0.6)	—
Anaemia	476 (8.7)	148 (11.0)	1.21 [0.99–1.47]	15 (5.9)	0.95 [0.55–1.62]	48 (9.4)	1.23 [0.89–1.68]	63 (14.1)	1.38 [1.04–1.85]	34 (10.4)	1.52 [1.05–2.21]
OR per −1 g/dL Hb mean (SD)^a^	11.4 (1.03)	11.3 (1.03)	1.05 [0.98–1.11]	11.6 (1.05)	0.98 [0.86–1.12]	11.4 (1.02)	1.04 [0.95–1.15]	11.3 (1.13)	0.99 [0.90–1.10]	11.5 (0.98)	1.03 [0.92–1.16]

*Note*: ORs adjusted for age, year of birth, GP record abstraction status.

Abbreviations: GP, general practitioner; HT, hypertension.

^a^ Lowest value of haemoglobinemia recorded in the obstetric record during the pregnancy.

**TABLE 3:** Associations between maternal infections during pregnancy and other pregnancy‐related illnesses, and childhood cancer: ALL, AML, HL, NHL and gliomas.

	ALL (*N* = 1130)		AML (*N* = 194)		NHL (*N* = 178)		HL (*N* = 71)		Glioma (*N* = 273)	
	*n* (%)	OR [95% CI]	*n* (%)	OR [95% CI]	*n* (%)	OR [95% CI]	*n* (%)	OR [95% CI]	*n* (%)	OR [95% CI]
*Infections*										
Any	376 (33.3)	0.94 [0.81–1.09]	70 (36.1)	1.01 [0.74–1.39]	59 (33.1)	1.51 [1.08–2.13]	16 (22.5)	0.95 [0.52–1.71]	73 (26.7)	1.01 [0.75–1.35]
Urinary tract	72 (6.4)	0.97 [0.74–1.27]	11 (5.7)	0.84 [0.45–1.57]	21 (11.8)	2.36 [1.46–3.81]	3 (4.2)	—	17 (6.2)	1.10 [0.66–1.82]
Genital	59 (5.2)	0.83 [0.62–1.12]	18 (9.3)	1.48 [0.89–2.46]	8 (4.5)	0.96 [0.46–2.00]	1 (1.4)	—	14 (5.1)	1.06 [0.61–1.86]
*Other illnesses*										
Gestational HT or preeclampsia	118 (10.4)	1.10 [0.89–1.37]	19 (9.8)	1.03 [0.63–1.67]	28 (15.7)	1.70 [1.12–2.58]	4 (5.6)	—	30 (11.0)	1.16 [0.78–1.71]
Preeclampsia	30 (2.7)	1.09 [0.73–1.64]	4 (2.1)	—	12 (6.7)	2.71 [1.46–5.03]	1 (1.4)	—	10 (3.7)	1.44 [0.74–2.77]
Polyhydramnios	15 (1.3)	1.51 [0.84–2.71]	5 (2.6)	3.01 [1.18–7.70]	5 (2.8)	2.80 [1.09–7.19]	0 (0)	—	4 (1.5)	—
Vomiting or hyperemesis	171 (15.1)	0.97 [0.80–1.16]	29 (14.9)	0.92 [0.61–1.39]	21 (11.8)	0.92 [0.57–1.47]	13 (18.3)	1.60 [0.86–2.97]	43 (15.8)	1.22 [0.87–1.72]
Anaemia	112 (9.9)	1.07 [0.86–1.34]	33 (17.0)	2.07 [1.40–3.08]	9 (5.1)	0.76 [0.38–1.50]	6 (8.5)	1.66 [0.70–3.91]	19 (7.0)	0.92 [0.57–1.50]
OR per −1 g/dL Hb mean (SD)^a^	11.3 (1.01)	1.02 [0.95–1.09]	11.2 (1.16)	1.20 [1.03–1.39]	11.6 (1.00)	0.97 [0.82–1.13]	11.6 (1.19)	1.05 [0.81–1.34]	11.5 (0.99)	1.00 [0.88–1.14]

*Note*: ORs adjusted for age, year of birth, GP record abstraction status.

Abbreviations: GP, general practitioner; HT, hypertension.

^a^ Lowest value of haemoglobinemia recorded in the obstetric record during the pregnancy.

Evidence for association with gestational hypertension and/or preeclampsia is generally weak across all cancers, including leukaemia (Table 2). Preeclampsia alone was, however, associated with all lymphomas combined (OR = 2.03 [1.12–3.67]); the OR for NHL being 2.71 [1.46–5.03] (the numbers were too small to analyse HL separately).

Severe hyperemesis was more frequently recorded in mothers of ALL cases; the association decreasing slightly after excluding children with Down syndrome (OR = 2.50 [0.89–7.04]). Although the estimation was based on small numbers, polyhydramnios appeared more frequently in mothers of acute myeloid leukaemia (AML) and NHL cases than mothers of controls. No evidence for an association between diabetes (pre‐existing or gestational) and childhood leukaemia was found; the OR for gestational diabetes and leukaemia was 0.69 [0.30–1.57] (7 exposed cases). Anaemia during pregnancy was associated with AML (OR = 2.07 [1.40–3.08]), but not ALL; further, the OR of AML for each decrease in 1 g/dL haemoglobin showed a significant linear trend (OR = 1.20 [1.03–1.39], *p*_trend_ = 0.02; *p*_linearity_ = 0.33).

### 3.3 Associations with non‐haematological tumours

There was no evidence of an association between maternal infections in pregnancy and CNS or embryonal tumours, either overall, by cancer subtype or infection type (Tables 2, 3, 4). Likewise, neither gestational hypertension nor preeclampsia were found to be associated with solid tumours, although 5 of 22 mothers of hepatoblastoma cases had a record of either gestational hypertension or preeclampsia (OR = 2.83 [1.04–7.74], data not shown).

**TABLE 4:** Associations between maternal infections during pregnancy and other pregnancy‐related illnesses, and childhood cancer: Embryonal tumours (not showing hepatoblastoma due to small numbers).

	Medulloblastoma (*N* = 94)		Retinoblastoma (*N* = 72)		Neuroblastoma (*N* = 144)		Nephroblastoma (*N* = 137)		Embr. rhabdomyosarcoma (*N* = 71)	
	*n* (%)	OR [95% CI]	*n* (%)	OR [95% CI]	*n* (%)	OR [95% CI]	*n* (%)	OR [95% CI]	*n* (%)	OR [95% CI]
*Infections*										
Any	28 (29.8)	1.04 [0.65–1.67]	20 (27.8)	0.84 [0.48–1.46]	40 (27.8)	0.76 [0.51–1.12]	42 (30.7)	1.07 [0.72–1.59]	27 (38.0)	1.33 [0.79–2.22]
Urinary tract	6 (6.4)	1.07 [0.46–2.47]	5 (6.9)	1.07 [0.42–2.70]	8 (5.6)	0.81 [0.39–1.68]	10 (7.3)	1.21 [0.62–2.34]	3 (4.2)	—
*Other illnesses*										
Gestational HT or preeclampsia	10 (10.6)	1.12 [0.58–2.18]	4 (5.6)	—	7 (4.9)	0.49 [0.23–1.06]	15 (10.9)	1.17 [0.68–2.02]	6 (8.5)	0.88 [0.38–2.03]
Vomiting or hyperemesis	11 (11.7)	0.80 [0.42–1.52]	17 (23.6)	1.85 [1.05–3.26]	24 (16.7)	1.10 [0.69–1.73]	19 (13.9)	0.99 [0.60–1.63]	15 (21.1)	1.49 [0.83–2.68]
Anaemia	16 (17.0)	2.36 [1.36–4.11]	14 (19.4)	1.83 [1.01–3.33]	21 (14.6)	1.39 [0.86–2.25]	12 (8.8)	0.83 [0.45–1.52]	16 (22.5)	2.91 [1.64–5.16]
OR per −1 g/dL Hb mean (SD)^a^	11.3 (1.22)	1.20 [0.97–1.47]	11.3 (1.30)	0.93 [0.73–1.19]	11.4 (1.19)	0.87 [0.73–1.04]	11.3 (0.97)	1.01 [0.85–1.21]	11.1 (1.14)	1.34 [1.05–1.69]

*Note*: ORs adjusted for age, year of birth, GP record abstraction status.

Abbreviations: GP, general practitioner; HT, hypertension.

^a^ Lowest value of haemoglobinemia recorded in the obstetric record during the pregnancy.

Polyhydramnios was associated with embryonal tumours; this condition was recorded for 3 retinoblastoma (4.2%) and 2 hepatoblastoma (9.1%, data not shown) cases, with small numbers limiting further analyses.

Maternal vomiting or hyperemesis was recorded more frequently for mothers of retinoblastoma cases than controls. There was no evidence of association between maternal diabetes (pre‐existing or gestational) and CNS tumours.

Maternal anaemia during pregnancy was associated with a 2‐to‐3 times increased risk of medulloblastoma, retinoblastoma and embryonal rhabdomyosarcoma (Table 4), with a significant log‐linear dose–response relationship for embryonal rhabdomyosarcoma (*p*_trend_ = 0.02; *p*_linearity_ = 0.25) and borderline for medulloblastoma (*p*_trend_ = 0.08; *p*_linearity_ = 0.06).

### 3.4 Additional analyses

Additional adjustment for maternal age at birth, pregnancy order, UKCCS region, and deprivation quintile did not alter the results, although the association between polyhydramnios and NHL became weaker (OR = 2.37 [0.84–6.73]). Sensitivity analyses conducted with a 11 g/dL threshold for haemoglobin showed weakened associations with AML, medulloblastoma, retinoblastoma and rhabdomyosarcoma, with ORs ranging from 1.09 (retinoblastoma) to 1.90 (medulloblastoma). There was some evidence suggesting that the association between anaemia and childhood embryonal rhabdomyosarcoma could be stronger in very young children, with an OR of 8.60 [2.92–25.33] in children aged 0 to <2 years and an OR of 1.85 [0.86–3.97] in older children (aged 2–14 years) (LR test interaction *p* = 0.02).

## 4 DISCUSSION

Using information from obstetric and primary care records, we found maternal anaemia in pregnancy to be positively associated with childhood AML and three types of embryonal tumours, with results compatible with a log‐linear decreasing dose–response relationship of haemoglobin levels for AML and embryonal rhabdomyosarcoma. In addition, we found that both urinary tract infections and preeclampsia were positively associated with childhood NHL. No evidence supporting an increased risk of leukaemia following maternal infection during pregnancy was found. Although rare, polyhydramnios was recorded more frequently in pregnancies of mothers whose children developed AML or NHL.

One of the main strengths of the UKCCS is that information relating to the diagnoses of maternal illnesses during pregnancy came from medical records compiled contemporaneously, significantly limiting the possibility for misclassification of the exposures. While we cannot rule out the possibility that some women did not visit their healthcare professional whilst being ill during pregnancy, misclassification in relation to the exposures, if any, would be non‐differential. Furthermore, as only a few English regions did not abstract maternal medical records, and with most mothers consenting to medical record access, our analyses showed no obvious selection bias. We have previously shown that maternal report of infections in pregnancy has poor validity.^27^ Unlike maternal interviews, medical records provide information on prenatal care and maternal medical history that is recorded contemporaneously and is exempt from recall bias. In some countries, maternal health information is recorded on birth certificate; however, the accuracy of this has been questioned, and birth certificates are generally considered to have poor validity (reviewed by Northam and Knapp^32^).

Using data pooled from primary‐care and obstetric records, we benefitted from medical information collected in two healthcare settings. In the United Kingdom, primary care services are the cornerstone of healthcare organisation within the National Health Service (NHS). Access to care for expectant mothers is free, and antenatal care is organised by midwives and GPs who specialise in pregnancy care. GPs remain the point of contact for all non‐pregnancy related health care issues, and as expected, we observed that general health issues, such as common infections, were more commonly recorded in GP records, whereas information on pregnancy‐related illnesses including hypertensive disorders, polyhydramnios and gestational diabetes, were more commonly recorded in obstetric records. Although this article did not aim to directly compare the two data sources, it is interesting to note that the results from primary‐care and obstetric records were generally similar.

The association between childhood cancers and maternal infections in pregnancy have been assessed in many types of study; ecological, cohort, and case–control, but there is still no consensus as to whether any specific maternal infection in pregnancy is associated with the risk of childhood cancer. In this regard, urinary and genital tract infections, along with some specific viral infections such as influenza and varicella (chickenpox), have been the most studied.^33^ Influenza has been reported to be positively associated with all childhood cancers combined,^34^ all leukaemias, and ALL,^35^ with weaker associations observed for neuroblastoma^3^ and Wilms' tumour^36^ in several case‐control studies based on maternal report. However, in agreement with other reports,^37^ including an international cohort study,^8^ our findings did not support a positive association with acute leukaemia. The small numbers of retinoblastoma and hepatoblastoma cases whose mothers were diagnosed with influenza during pregnancy (one hepatoblastoma and two retinoblastoma cases) hampered interpretation. In our study, only 23 control mothers and 12 case mothers had a record of chickenpox during pregnancy, and so we could not examine this association.

Genital infections are comparatively frequent in pregnancy, and their potential association with childhood cancer has been investigated in different ways, with some studies focusing on sexually transmitted diseases,^6^, ^35^, ^38^ and others on vaginal infections, vaginitis^1^, ^3^, ^4^, ^8^, ^36^, ^38^ or herpes.^17^, ^39^ One study using maternity charts,^4^ reported associations between maternal lower genital tract infection in pregnancy and childhood ALL and AML (ORs 1.63 [1.04–2.53] and 4.00 [0.85–18.8] respectively); which was also seen for any childhood leukaemia in a recent cohort analysis of hospital‐based records (HR = 2.42 [1.50–3.92]),^9^ but not in a pooled cohort study, based on self‐reported data.^8^ Inconsistent results were also reported with neuroblastoma,^3^, ^38^ central nervous system tumours,^10^, ^39^ and while two studies have reported a positive association between vaginal infections in pregnancy and Wilms' tumour,^1^, ^36^ our analyses did not replicate this finding.

Apart from a positive association with non‐Hodgkin lymphoma, we found no associations with urinary tract infections, which is in line with most previous studies reporting no significant association with neuroblastoma,^38^ childhood leukaemia^4^, ^17^, ^35^, ^37^ and leukaemia in children with Down's syndrome,^40^, ^41^ although an increased risk of childhood leukaemia was reported in cohort studies.^8^, ^9^ Regarding non‐Hodgkin lymphoma, there is only one previous publication, a health registry linkage study, which reported no association between NHL and urinary tract infections.^42^

Anaemia in pregnancy and the risk of childhood cancer has frequently been examined, with the majority of studies relying on self‐report or birth certificates, showing no or weak associations with leukaemia and other types of childhood cancers. A positive association was however reported for neuroblastoma in two studies,^13^, ^14^ and for childhood leukaemia in three,^11^, ^12^, ^14^ including one based on self‐report. It is unclear how much recall bias affected the studies relying on self‐reported anaemia.^26^, ^43^ Based on medical records, our analyses showed increased odds of childhood AML and several types of embryonal tumours in children born to mothers with anaemia during pregnancy, with evidence of an inverse dose–response relationship with haemoglobin level. As expected, when using a broader definition of anaemia in pregnancy (Hb < 11 g/dL), the associations were weakened. The observed proportion of pregnant women with anaemia in our study (28.6% based on the 11 g/dL definition) was in agreement with the prevalence estimated in 1993–2005 in Europe (25.1% [95%CI 18.6%–31.6%]) and in the UK (15.2% [95%CI 3.8%–44.7%]).^44^ In a previous report, we found positive associations between iron prescription during pregnancy and ALL, medulloblastoma and nephroblastoma, but not AML (OR = 1.26 [0.86, 1.85]).^31^

Our observation that maternal anaemia during pregnancy could be associated with AML and embryonal tumours, which are typically diagnosed in infancy and early childhood, supports an in utero pathological mechanism, rather than a chance finding. It is however unclear whether maternal anaemia could be a risk factor for these malignancies in itself, a marker, or an intermediate factor. While it would have been interesting to investigate the association with anaemia by trimester, analyses were limited not only by small numbers but also by the fact that blood tests are performed less frequently in trimesters 1 and 2. Maternal anaemia in pregnancy has been reported to be associated with poor pregnancy outcomes, including prematurity, low birthweight and foetal distress, and severe maternal anaemia is thought to impair foetal haemoglobin levels.^45^ We could therefore hypothesize that this would in turn result in an abnormally deep foetal hypoxia. Hypoxia is involved in normal embryogenesis and haematopoiesis and through the effects of the hypoxia‐induced factors (HIF).^46^ Under hypoxic conditions, HIF‐1 and HIF‐2 regulate the transcription of hundreds of genes (up‐ or down‐regulation), including erythropoietin (EPO), vascular endothelial growth factor (VEGF), transferrin and transferrin receptor.^46^ The HIF‐1α subunit has been shown to be involved in erythropoiesis, B lymphocyte development, T lymphocyte differentiation and haematopoiesis in general.^47^ Moreover, hypoxia has been shown to promote neuroblastoma cells dedifferentiation into stem cell‐like phenotype and has been suggested to promote the tumour development itself.^48^ Alternatively, maternal anaemia could be a proxy of an unknown causal factor, or an intermediate factor, in the pathway of the development of childhood malignancies. In high‐income countries, the predominant cause of anaemia in pregnancy is nutritional deficiency, mainly of iron and folic acid.^45^ Anaemia can also be caused by haemoglobinopathies and other causes of chronic inflammation, such as chronic infection and cancer, but these are unlikely to explain the relatively high frequency of anaemia observed in the UKCCS. Previous studies reported an inverse association between folic acid supplementation in pregnancy and several types of childhood cancer, including leukaemia and neuroblastoma.^49^ More research is needed to further examine the relationship between maternal anaemia and malignancies in early childhood.

In summary, this study provides additional evidence that maternal anaemia could be positively associated with childhood AML and embryonal tumours. Interpretation regarding associations with preeclampsia, polyhydramnios and severe hyperemesis warrant cautious interpretation due to low numbers. There was little evidence that any maternal infections occurring in pregnancy, including influenza, were associated with childhood cancer; the association with urinary tract infections requires further study. Additional research is needed to clarify the potential aetiological role of maternal anaemia, iron and folate deficiency in the pathogenesis of childhood leukaemia and embryonal tumours.

## AUTHOR CONTRIBUTIONS

**Audrey Bonaventure:** Conceptualization; formal analysis; funding acquisition; methodology; visualization; writing – original draft, writing – review & editing. **Jill Simpson:** Conceptualization; data curation; methodology, writing – review & editing. **Eleanor Kane:** Methodology, writing – review & editing. **Eve Roman:** Conceptualization; data curation; funding acquisition; methodology; supervision, writing – review & editing.

## CONFLICT OF INTEREST STATEMENT

The authors declare no conflicts of interest.

## ETHICS STATEMENT

This study involves human participants and was approved by Yorkshire & the Humber‐Leeds West Research Ethics Committee (reference 18/YH/0135). Mothers consented to participate in the study and following being interviewed, their consent was sought to abstract information from their primary care and obstetric medical records.

## Supporting information


**Data S1.** Supplementary Tables.

## Data Availability

UKCCS data are contributing to several ongoing research projects. For information on how to collaborate with UKCCS researchers and investigate questions of interest, or for further information, please email the Principal Investigator (ER).
