# Supplementary material for: Maternal illnesses during pregnancy and the risk of childhood cancer: A medical‐record based analysis (UKCCS)
Source: Int J Cancer. 2024 Nov 13;156(5):920–9. doi: 10.1002/ijc.35166 (PMC11701404; doi:10.1002/ijc.35166)
Supplement: Supplementary file 1 — Data S1. Supplementary Tables. [file IJC-156-920-s001.pdf]

__Supplementary to:__

Maternal illnesses during pregnancy and the risk of childhood cancer: a medical-record based analysis (UKCCS)

Audrey Bonaventure, Jill Simpson, Eleanor Kane, Eve Roman

Supplementary table 1: Classification used for maternal illnesses, based on ICD-10 codes

Supplementary table 2: Description of participants included in the UKCCS and those included in the analyses by type of medical record abstracted

Supplementary table 1: Classification used for maternal illnesses, based on ICD-10 codes

**Disease category**	**ICD-10 codes**
Any infection (definite)	A00-B99, E06.0, E32.1, G00-G08, H00.0, H05.0, H60, H66, I88, I89.1, J00-J06, J09-J18, J20-J22, J32, J34.0, J35.0, J36, J40, K02.9, K04.6, K04.7, K05.6, K11.2, K11.3, K12.0, K12.2, K37, K81, L00-L03, L05, L08, N10-N12, N30.9, N39.0, N61, N70, N72, N73.9, N75-N76, O23, O86, O91, T81.4.
Urinary tract infection: Cystitis or kidney infections	N10, N12, N30.9, N39.0, O23.0, O23.1, O23.2, O23.4, O23.9
Genital infection: Vaginitis, genital candidiasis or trichomoniasis	N76.0, B37.3, A59.9
Influenza	J10, J11
Gestational HT, preeclampsia or eclampsia<br>Preeclampsia or eclampsia	O13, O14, O15<br>O14, O15
Polyhydramnios	O40
Any vomiting or hyperemesis<br>Severe hyperemesis	O21, R11<br>O21.1
Diabetes<br>Gestational diabetes	E10-E14, O24<br>O24.4
Anaemia	D50, D51, D52, D53, D55, D56, D57 (except D57.3), D58, D59, D60, D61, D62, D63, D64, O99.0

The code list might omit other codes absent from the data.

Supplementary table 2: Description of participants included in the UKCCS and those included in the analyses (with maternal medical record) by type of medical record abstracted

	Controls				Cases			
		Maternal medical record				Maternal medical record		
	**Interview* N (%)**	**Any N (%)**	**GP record N (%)**	**Obstetric N (%)**	**Interview* N (%)**	**Any N (%)**	**GP record N (%)**	**Obstetric N (%)**
**Total**	**7586**	**5499**	**2521**	**5169**	**3812**	**2885**	**1623**	**2721**
**Sex**								
Female	3327 (43.9)	2431 (44.2)	1117 (44.3)	2290 (44.3)	1673 (43.9)	1274 (44.2)	728 (44.9)	1207 (44.4)
Male	4259 (56.1)	3068 (55.8)	1404 (55.7)	2879 (55.7)	2139 (56.1)	1611 (55.8)	895 (55.1)	1514 (55.6)
**Age (years)**								
1st year of life	627 (8.3)	490 (8.9)	215 (8.5)	477 (9.2)	314 (8.2)	256 (8.9)	139 (8.6)	250 (9.2)
1-4	3171 (41.8)	2402 (43.7)	1142 (45.3)	2300 (44.5)	1594 (41.8)	1242 (43.1)	739 (45.5)	1198 (44.0)
5-9	2065 (27.2)	1517 (27.6)	652 (25.9)	1435 (27.8)	1043 (27.4)	794 (27.5)	420 (25.9)	749 (27.5)
≥ 10	1723 (22.7)	1090 (19.8)	512 (20.3)	957 (18.5)	861 (22.6)	593 (20.6)	325 (20.0)	524 (19.3)
mean (sd)	5.6 (4.19)	5.3 (4.06)	5.3 (4.05)	5.2 (4.01)	5.6 (4.18)	5.4 (4.10)	5.2 (4.06)	5.2 (4.03)
**Maternal age at birth**								
< 25	2297 (30.3)	1617 (29.4)	719 (28.5)	1531 (29.6)	1252 (32.8)	934 (32.4)	498 (30.7)	885 (32.5)
25-29	2772 (36.5)	2015 (36.6)	904 (35.9)	1893 (36.6)	1383 (36.3)	1057 (36.6)	615 (37.9)	990 (36.4)
>29	2511 (33.1)	1867 (34.0)	898 (35.6)	1745 (33.8)	1175 (30.8)	894 (31.0)	510 (31.4)	846 (31.1)
missing	6 (0.1)				2 (0.1)			
mean (sd)	27.4 (5.17)	27.5 (5.15)	27.7 (5.19)	27.5 (5.18)	27 (5.18)	27.0 (5.15)	27.2 (5.16)	27.0 (5.16)
**Pregnancy order**								
1	2625 (34.6)	1885 (34.3)	841 (33.4)	1782 (34.5)	1324 (34.7)	1004 (34.8)	549 (33.8)	951 (35.0)
2	2471 (32.6)	1815 (33.0)	848 (33.6)	1688 (32.7)	1266 (33.2)	934 (32.4)	520 (32.0)	881 (32.4)
3	1377 (18.2)	993 (18.1)	470 (18.6)	939 (18.2)	676 (17.7)	515 (17.9)	291 (17.9)	491 (18.0)
4+	1113 (14.7)	806 (14.7)	362 (14.4)	760 (14.7)	546 (14.3)	432 (15.0)	263 (16.2)	398 (14.6)
**Deprivation**								
(affluent) 1	1406 (18.5)	1027 (18.7)	528 (20.9)	963 (18.6)	671 (17.6)	506 (17.5)	313 (19.3)	473 (17.4)
2	1529 (20.2)	1152 (20.9)	553 (21.9)	1070 (20.7)	768 (20.1)	611 (21.2)	323 (19.9)	575 (21.1)
3	1610 (21.2)	1230 (22.4)	560 (22.2)	1146 (22.2)	768 (20.1)	600 (20.8)	334 (20.6)	567 (20.8)
4	1498 (19.7)	1111 (20.2)	480 (19.0)	1056 (20.4)	776 (20.4)	586 (20.3)	342 (21.1)	564 (20.7)
(deprived) 5	1489 (19.6)	952 (17.3)	392 (15.5)	910 (17.6)	798 (20.9)	566 (19.6)	303 (18.7)	529 (19.4)
missing	54 (0.7)	27 (0.5)	8 (0.3)	24 (0.5)	31 (0.8)	16 (0.6)	8 (0.5)	13 (0.5)
**Cancer diagnoses**								
Leukaemia					1723 (45.2)	1347 (46.7)	897 (55.3)	1266 (46.5)
Lymphoma					350 (9.2)	254 (8.8)	115 (7.1)	236 (8.7)
CNS**					685 (18.0)	510 (17.7)	231 (14.2)	487 (17.9)
Embryonal**					578 (15.2)	446 (15.5)	228 (14.0)	422 (15.5)
Other					476 (12.5)	328 (11.4)	152 (9.4)	310 (11.4)

* excluding 20 cases and 29 controls whose biological mother could not be interviewed

** medulloblastoma included in the group of CNS tumours
